# What is the impact on recruitment of a shortened compared with a standard-length participant information leaflet? PROMETHEUS in IBD-BOOST: study within a trial, a decentralised UK randomised controlled trial

**DOI:** 10.1186/s13063-025-08931-6

**Published:** 2025-06-18

**Authors:** L. Miller, A. Hart, F. Cléirigh-Büttner, C. Arundel, T. Hamborg, S. McGuinness, R. Moss-Morris, A. Parker, C. Relton, I. Stagg, L. Sweeney, V. Wileman, Z. Zenasni, C. Norton

**Affiliations:** 1 Unit for Social and Community Psychiatry, Centre for Psychiatry and Mental Health (CPMH), Wolfson Institute of Population Health, Queen Mary University of London, Yvonne Carter Building, 58 Turner Street, London, E1 2AB UK; 2 St Mark’s Hospital, Acton Lane, Central Middlesex, London, NW10 7NS UK; 3 Pragmatic Clinical Trials Unit, Centre for Evaluation and Methods, Wolfson Institute of Population Health, Queen Mary University of London, Yvonne Carter Building, 58 Turner Street, London, E1 2AB UK; 4 York Trials Unit, Department of Health Sciences - Faculty of Science, ARRC Building, University of York, York, YO10 5DD UK; 5 Bristol Vaccine Centre, University of Bristol, 39-41 St Michaels Hill, Bristol, BS2 8DX UK; 6 Department of Psychology, Institute of Psychiatry, Psychology and Neuroscience, King’s College London, Guy’s Hospital Campus, 5 Floor Bermondsey Wing, London Bridge, London, SE1 9RT UK; 7 NIHR Royal Free Clinical Research Facility, Royal Free London NHS Foundation Trust, Royal Free Hospital, Pond Street, London, NW3 2QG UK; 8 Institute of Psychiatry, Psychology and Neuroscience, King’s College London, De Crespigny Park, Denmark Hill, London, SE5 8AB UK; 9 School of Public Health, Imperial Clinical Trials Unit, Imperial College London, Stadium House, White City Campus, 80 Wood Lane, London, W12 7TA UK; 10 Florence Nightingale Faculty of Nursing, Midwifery and Palliative Care, King’s College London, 57 Waterloo Road, London, SE1 8WA UK

**Keywords:** SWAT, Study within a trial, Recruitment methods, Research methods, Trial design, Participant Information Leaflet, PIL, Embedded randomised controlled trial, Ethics, Informed consent, Clinical trials

## Abstract

**Background:**

Participant Information Leaflets (PILs) are lengthy and increasingly complex, and could deter research participation. A shortened PIL may be more appealing as it is likely to provide a more a manageable volume of information. Previous research has found that shortened PILs are no less effective for recruitment outcomes, and we deemed it useful to replicate this in an online setting. We also decided to compare retention rates, given the potential for more information to increase participants’ motivation.

**Aim:**

To evaluate the efficacy of a shortened vs standard-length PIL on trial recruitment and retention rates.

**Methods:**

This two-arm study within a trial (SWAT) was embedded in a host randomised controlled trial (RCT)—IBD-BOOST.

Potential participants were randomised to receive a standard-length or shortened PIL electronically for recruitment to the host RCT. An ethics committee approved potential participants being blinded to this randomisation.

Primary outcome: The percentage of SWAT participants receiving the shortened vs standard PIL who were recruited to the RCT.

**Results:**

Four thousand two hundred one participants were randomised to the standard-length (*n* = 2099) and shortened (*n* = 2102) PIL arms. Thirty-four email queries were received about the PILs—18 from those who received the standard and 16 from those receiving the shortened. Seven hundred eight SWAT participants were recruited to the RCT—333 (15.86%) who received the standard-length PIL and 375 (17.84%) who received the shortened (OR = 1.15, (95%CI = 0.98, 1.35), *p* = 0.09). Retention rates in the RCT were not statistically different between groups.

**Conclusion:**

We did not find evidence of a significant difference between RCT recruitment and retention rates in participants randomised to the standard-length PIL compared with the shortened. It may be that a shortened PIL has the same effect on recruitment and retention outcomes, providing that the trial does not require extensive information for other reasons (e.g. safety). Therefore, it could be argued that researchers have a choice about how long to make PILs, perhaps with a link to more detail. Alternatively, given that there was no benefit of the shortened PIL, it may be worth comparing written PILs with other methods of conveying information to determine optimal means of encouraging participation and retention in decentralised trials.

**Host trial registration:**

A randomised controlled trial of supported, online, self-management for symptoms of fatigue, pain and urgency/incontinence in people with inflammatory bowel disease: the IBD-BOOST trial (ISRCTN71618461 on ISRCTN registry).

**Supplementary Information:**

The online version contains supplementary material available at 10.1186/s13063-025-08931-6.

## Background

Participant Information Leaflets (PILs) are often lengthy and increasingly complex—regularly eight pages long [1]—despite the current UK Health Research Authority (HRA) guidance in England advising a proportionate approach. They recommend that PILs should not provide too much detail but rather ensure that a clear and concise picture of the research is given, explaining the purpose of and background to the research and invitation, what taking part would involve, and the benefits and disadvantages of participating [2]. Perhaps longer PILs have become habit, or are a result of researchers wanting to cover all bases and not leave anything out, but it has previously been argued that lengthy participant information deters people from reading it [3] and may act as a barrier to otherwise interested and eligible participants partaking in research [4].

An RCT comparing an interactive electronic PIL (where participants could choose both the type and level of detail accessed) with a standard-length electronic PDF copy of the PIL identified that only 9% of participants in the interactive arm accessed the available, more detailed information presented [5]. A shortened PIL may be more appealing to participants as it likely provides a more manageable volume of information to efficiently process and comprehend, which may encourage eligible participants to subsequently enrol in a trial [4]. Alternatively, written PILs may not be the best method for conveying information and alternative approaches, such as staging information [6] and/or using multimedia [7], might be more effective.

A Cochrane review of recruitment interventions in 2018 identified two trials that have evaluated two postal PILs—one short and one full-length [4, 8, 9]—and concluded moderate grade evidence that a shortened postal PIL makes little-to-no difference to recruitment outcomes compared with a full PIL (RD = 0%, 95% CI =  − 2% to 2%). In an online setting previous research has found that, when presented with three levels of study information to read (the first containing less than might be found on a standard PIL, the second corresponding to a standard PIL, and the third containing more information than a standard PIL) that most eligible participants chose to read the minimum (i.e. only the first of three levels of) information provided [10].

Decentralised clinical trials (DCTs) delivered remotely are an increasingly common and acceptable form of generating research evidence following adaptations during the COVID-19 pandemic [11], particularly using digitally enabled approaches [12]. Internet-based trials have the advantage of a wider reach and large diverse samples, as well as reduced costs and increased convenience for participants [13]. All of these can be challenges when recruiting to a study, and low rates of recruitment and retention have impacts on reliability, generalisability, statistical certainty, resource waste and access to potentially effective treatments [14].

Within our DCT, we deemed it useful to run a SWAT replicating comparing recruitment outcomes between shortened and standard-length online PIL arms. We also decided to compare retention between SWAT arms, given the potential for people who have read more information (by being allocated to the full-length PIL) to be more motivated to remain in the trial as they are better primed about what to expect.

## Objectives

To evaluate whether a shortened PIL improves trial recruitment and retention outcomes compared with a standard-length PIL in the IBD-BOOST host RCT.

## Methods

A two-arm SWAT was performed with an allocation ratio of 1:1 (shortened vs standard-length PIL). This SWAT was prioritised by the PROMETHEUS programme (https://www.york.ac.uk/healthsciences/research/trials/swats/prometheus/), which aimed to rapidly increase the evidence base around recruitment and retention strategies with SWAT evidence, funded by the Medical Research Council (MRC) and sponsored by the University of York. This SWAT was embedded in a UK National Institute of Health Research (NIHR) (RP-PG-0216–20001) RCT of a supported, online, self-management intervention for symptoms of fatigue, pain, and faecal urgency/incontinence in people with inflammatory bowel disease (IBD)—the IBD-BOOST trial (Trial registration: ISRCTN71618461) [15]. This was a two-arm, parallel-group, RCT recruiting patients from clinics and national registries.

This SWAT was granted favourable ethical opinion by the National Research Ethics Committee & Health Research Authority (HRA) (London—Surrey Research Ethics Committee/19/LO/0750) as part of the ethical approval for the host RCT, including permission not to inform participants that they were randomised in the SWAT, once a shortened version of the HRA’s General Data Protection Regulation (GDPR) transparency wording was added.

### Participants

Potential participants for the SWAT included all patients identified from a preceding IBD-BOOST survey [16] of people with IBD, e.g. Crohn’s disease or ulcerative colitis, who (i) self-reported the impact of one or more symptoms of fatigue, pain, or urgency/incontinence on quality of life as 5 or more on a 0–10 scale, (ii) expressed a desire for treatment of their symptoms, and (iii) were thus eligible for the IBD-BOOST trial. There were no additional inclusion or exclusion criteria for the SWAT. Survey participants were recruited from the following sources:

- Unselected cohort of adults with IBD who attended one of 17 participating IBD clinics which had a register of all patients recorded on a database
- Unselected patient members of the charity Crohn’s & Colitis UK (CCUK)
- Patients with IBD who had previously been recruited to the UK National IBD BioResource register
- Self-selected via social media (Twitter and Facebook accounts of CCUK and the study team) and IBD-related websites (such as CCUK)

Inclusion criteria for the previous IBD-BOOST survey were:

- A diagnosis of Crohn’s disease, ulcerative colitis, or another type of IBD
- 18 years old and over
- Living in England, Scotland or Wales
- Able to give informed consent

SWAT data were collected using REDCap, which is a secure online application for both building and managing randomisation and study databases. Using the REDCap study database the research team randomised eligible IBD-BOOST Survey participants into one of the two SWAT arms, and then sent out trial invitations with a link to the corresponding PIL and electronic consent form via email.

Two ‘reminder’ notifications were sent to non-responders via email and text after 10 and 20 days. For the main RCT, consent forms and questionnaires at baseline, 6, and 12 months were completed remotely by participants using an electronic link. Only 532 participants were sent a 12-month assessment due to delays resulting from the COVID-19 pandemic. Two ‘reminder’ notifications were sent to non-responders via email and text after 10 and 20 days.

### Interventions

SWAT participants were randomised to one of the following interventions, which they accessed online:

1. A standard-length, online PIL, with all UK National Research Ethics Service (NRES) required details provided in a single document

The standard-length PIL was developed by the IBD-BOOST team following NRES guidance. The content of the standard PIL included general information about the purpose of the RCT, how and why the participant might be involved, key trial concepts such as randomisation, the intervention being assessed, and potential risks and benefits of the intervention, the participant’s right to withdraw, trial team contact information, confidentiality information, and details on who was funding and monitoring the research. The standard PIL was four A4 pages long.

2. A shortened, online PIL

The shortened PIL comprised a single, online, A4 page of text with a concise summary of the IBD-BOOST RCT. The shortened PIL had less detail about the steps of the study, and omitted text about the organisations involved, confidentiality, information management and data sharing procedures, plans for dissemination, the organisers and funders of the research, and information about the Research Ethics Committee (REC) approval. At the end of the shortened PIL was an accessible hyperlink to the standard-length PIL.

The information in both PILs was reviewed by the IBD-BOOST PPI group and PROMETHEUS PPI panels, the IBD-BOOST Trial Steering Committee (TSC), and the London—Surrey REC. Both PILs were presented in electronic Portable Document Format (PDF) when sent digitally, and the accompanying cover email/letter template was also on a single A4 page.

### Outcomes

#### Primary outcome

1. The percentage of SWAT participants receiving the shortened compared with the standard PIL who consented to and were randomised into the IBD-BOOST host RCT.

#### Secondary outcomes

1. The number of follow-up queries received by the study team prior to randomisation to the IBD-BOOST RCT.
2. Six- and 12-month retention rates within the IBD-BOOST RCT.

### Sample size calculation

The sample size calculation for the IBD-BOOST RCT has been outlined in the main trial protocol [15] and paper. The trial’s target sample size was a minimum of 740 participants to be randomised across two arms. As is usual with a SWAT, we did not undertake a formal statistical power calculation to determine the study sample size since the sample size was constrained by the number of patients being approached in the IBD-BOOST host RCT. Thus, the sample size was the total number of patients invited into the IBD-BOOST host trial.

### Randomisation: sequence generation

The PIL version sent to each participant was determined by random allocation. Eligible participants were randomised using a 1:1: ratio, stratified by their entrance pathway (i.e. whether they entered via an IBD-BOOST Optimise medical management study following their participation in IBD-BOOST Survey, or via a direct entry route, see Appendix 1: Flow Chart). Blocked randomisation with randomly varying block sizes of 4 and 6 was used.

### Allocation concealment mechanism

Concealed allocation lists were generated by an independent statistician and uploaded to the REDCap randomisation system. They were accessed only by staff sending RCT invitations to eligible participants.

### Implementation

Participants were sent an invitation link via email after the research team had entered participants’ ID, date of consenting to participate in the IBD-BOOST survey, National Health Service (NHS) site, and entrance pathway into the REDCap system. Sent invitations were automatically recorded by the system.

### Blinding

Participants were not aware that they were part of a trial evaluating a recruitment intervention and so were blind to the SWAT hypothesis, as is routine for MRC-funded PROMETHEUS studies.

### Statistical methods

Arm-level frequencies (n) and percentages (%) are reported for categorical outcomes. Arm-level means and standard deviations (SD) are reported for continuous outcomes. Logistic regression was performed to estimate the unadjusted odds ratio (OR) comparing the odds of being randomised in the IBD-BOOST RCT amongst participants in the shortened and standard PIL arms of the SWAT. Logistic regression was also performed to estimate adjusted and unadjusted odds ratios comparing the odds of retention in the IBD-BOOST RCT 6 months and 12 months post-randomisation amongst participants in the shortened and standard PIL arms of the SWAT. IBD-BOOST RCT trial arm was adjusted for in multivariable logistic regression analyses. Ninety-five per cent confidence intervals (95% CIs) were constructed around OR point estimates to report the precision of the estimate. Participants were analysed according to the SWAT arm to which they were initially randomised irrespective of which PIL(s) they ultimately accessed, thus following the intention-to-treat principle. Hypothesis tests were two-tailed and the alpha level was set at 0.05.

Anonymised data from this SWAT will ultimately be combined in a meta-analysis with data from similar host RCTs participating in PROMETHEUS, in accordance with the PROMETHEUS data sharing agreement (https://www.york.ac.uk/healthsciences/research/trials/swats/prometheus/). Study results are presented in accordance with the Trial Forge Guidance 4 for SWATs [17]. The checklist is presented in Supplementary online material 1.

## Results

**Fig. 1:**
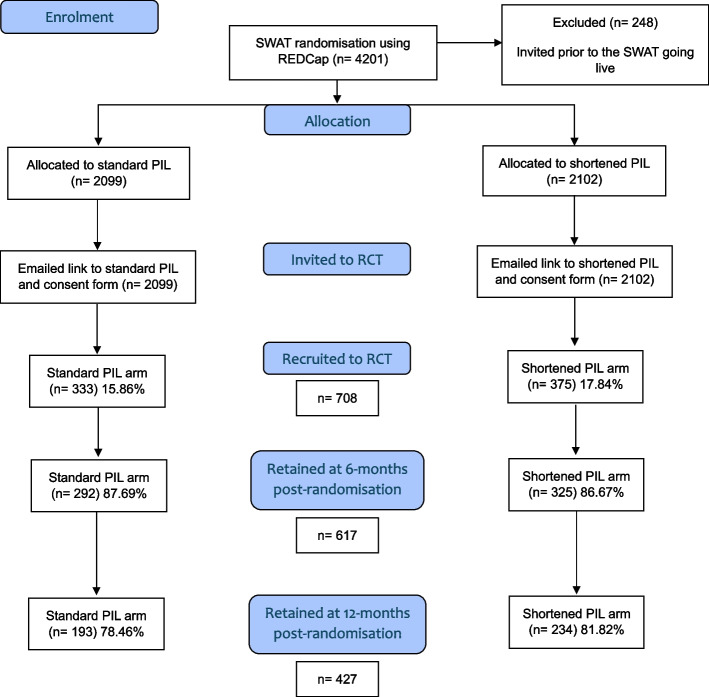
Participant flow through the IBD-BOOST SWAT and RCT

Participant flow is shown in Fig. 1. Two hundred forty-eight participants were invited to the RCT before SWAT randomisation, and PILs were live online and so were excluded.

### Recruitment

Trial recruitment took place between 27th August 2020 and 20th July 2022, with randomisation into the IBD-BOOST RCT finishing on 31 st July 2022.

6-month post-randomisation assessments started on the 20th July 2020, with both 6- and 12-month post-randomisation assessments completed on 24th March 2023. Only 532 of the participants were sent a 12-month assessment due to delays resulting from the COVID-19 pandemic.

### Baseline data

Baseline demographic and clinical characteristics for each arm of the SWAT are presented in Table 1.

**Table 1:** Baseline demographic and clinical characteristics in control and intervention arms of the SWAT

	**Standard PIL** **(*n* = 2099)**	**Shortened PIL** **(*n* = 2102)**	**Total** **(*N* = 4201)**
**IBD diagnosis, *n* (%)**			
Crohn’s disease	1117 (53.22%)	1132 (53.85%)	2249 (53.53%)
Other IBD	971 (46.26%	959 (45.62%)	1930 (45.94%)
Missing	11 (0.52%)	11 (0.52%)	22 (0.52%)
**Age, mean (SD)**	48.21 (14.66)	48.75 (14.78)	48.48 (14.72)
Missing, *n* (%)	5 (0.24%)	8 (0.38%)	13 (0.31%)
**Gender, *n* (%)**			
Female	1374 (65.46%)	1350 (64.22%)	2724 (64.84%)
Male	714 (34.02%)	740 (35.20%)	1454 (34.61%)
Prefer not to say	1 (0.05%)	1 (0.05%)	2 (0.05%)
Prefer to self-describe	4 (0.19%)	3 (0.14%)	7 (0.17%)
Missing	6 (0.29%)	8 (0.38%)	14 (0.33%)
**Ethnicity, *n* (%)**			
White	1985 (94.57%)	2003 (95.29%)	3988 (94.93%)
Mixed	32 (1.52%)	29 (1.38%)	61 (1.45%)
Asian	48 (2.29%)	42 (2.00%)	90 (2.14%)
Black	8 (0.38%)	7 (0.33%)	15 (0.36%)
Other	15 (0.71%)	12 (0.57%)	27 (0.64%)
Prefer not to say	6 (0.29%)	0 (0.00%)	6 (0.14%)
Missing	5 (0.24%)	9 (0.43%)	14 (0.33%)

Mean and standard deviation (SD) for continuous variables indicated by parentheses around SD; absolute frequencies and column-wise percentages for categorical data indicated by ‘%’ for percentage summary data

*Std*, standard; *PIL*, Participant Information Leaflet

### Numbers analysed

Four thousand two hundred one participants were invited to participate in the IBD-BOOST RCT and thus included in the SWAT. There were 2099 and 2102 participants who were randomly assigned to the standard and shortened PIL arms of the SWAT, respectively.

### Outcomes and estimation

There was very weak evidence of a difference in randomisation to the IBD-BOOST RCT between participants in shortened and standard PIL arms of the SWAT. Specifically, compared with participants in the standard PIL arm, participants in the shortened PIL arm demonstrated 1.15 times the odds of being randomised to one of the arms in the IBD-BOOST RCT (OR = 1.15, (95%CI = 0.98, 1.35), *p* = 0.09). This equates to an absolute difference of approximately 2% more participants in the shortened PIL arm being randomised to the IBD-BOOST RCT compared with the standard PIL arm (absolute percentage difference = 1.98% (95% CI =  − 0.29%, 4.24%)).

There was negligible evidence of a difference in trial retention rates at 6- and 12-month post-randomisation between participants randomised to shortened and standard PIL arms of the SWAT. Table 2 presents adjusted and unadjusted results for recruitment/randomisation and retention outcomes.

**Table 2:** Effect of randomly assigned shortened versus standard-length PIL on trial recruitment and retention outcomes (*N* = 4201)

Outcome	Standard PIL (*n* = 2099)		Shortened PIL (*n* = 2102)		Effect estimate (OR)	95% CI		*P*-value
	***n***	**%**	***n***	**%**				
**Recruited and randomised**	333	15.86%	375	17.84%	1.15	0.98	1.35	0.09
**Retained at 6 months post-randomisation**								
Unadjusted	292	87.69%	325	86.67%	0.91	0.59	1.42	0.69
Adjusted*					0.94	0.60	1.47	0.77
**Retained at 12 months post-randomisation**								
Unadjusted	193	78.46%	234	81.82%	1.20	0.89	1.63	0.23
Adjusted^*^					1.22	0.90	1.65	0.20

*PIL* Participant Information Leaflet, *OR* odds ratio, *95% CI* 95% confidence interval

^*^Adjusting for randomly assigned trial arm

The cost of an additional randomisation database and additional statistician time to administer this and analyse the results was approximately £4962, which was covered by the PROMETHEUS programme. Additional research staff time to conduct the SWAT, consult PPI, and write up these results has not been calculated. As we did not detect an effect between SWAT arms, cost per participant recruited or retained was not derived, but the costs of implementing either PIL would be the same as all PILs were online and no printing costs were incurred.

### Ancillary analyses

The study team received 34 email queries after SWAT randomisation: 18 from those who received the standard PIL and 16 from participants who received the shortened PIL, indicating no additional workload engendered by the shortened PIL.

As the format of the consent process was automated (using a single invitation link to proceed through the participant information and onto the form) it was not possible to measure the frequency and percentage of participants in each SWAT arm who expressed an interest in participating in IBD-BOOST, other than looking at the number in each arm who consented to participate.

Data on the number of shortened PIL participants clicking through to the standard PIL was deleted in error by the host university after the SWAT was completed and the PIL removed from the website and thus could not be analysed.

## Harm

No harm or unintended effect data were collected.

## Discussion

We found no difference in recruitment or retention outcomes between participants who accessed shortened and standard-length online PILs. These results align with a previous Cochrane review [8] that included two studies of shortened versus longer PILs sent by post [4, 9]. There was also no apparent difference in follow-up queries received (18 for the full-length vs 16 for the shortened PIL). With regard to the SWAT sample demographic characteristics, 2724 (64.84%) were female, the mean age was 48 years, and 3,988 (94.93%) were white. This is representative of the host trial population, where 524 (67.18%) were female, the mean age was 49 years, and 744 (95.38%) were white. The ethnicity of host trial participants was less diverse than the UK general population but aligns with previous research identifying a higher prevalence of IBD in people declaring their ethnicity as white [18].

## Limitations

This SWAT possesses limitations in that we were unable to answer two of our original secondary outcomes. Firstly, as the format of the consent process was automated (using a single invitation link to proceed through the participant information and onto the form) it was not possible to determine the proportion of patients in each SWAT arm who expressed an interest in participating in the IBD-BOOST RCT. Without directly recording an expression of interest, these data could not be derived other than observing the number of participants in each SWAT arm who participated. Secondly, we were unable to ascertain the number of participants in the shortened PIL arm who accessed the full PIL information because webpage activity data was deleted in error by the host university after the SWAT was completed and the information sheet removed from the website. Additionally, this SWAT was unable to evaluate whether participants recruited using a shortened (vs standard-length PIL) are more or less adequately informed from a clinical trial standpoint e.g. what do patients remember having consented to. Further studies, such as one conducted by researchers in the Netherlands [19], are needed to evaluate patients’ recollection and understanding of the information that they were given and the consents they gave.

## Generalisability

This SWAT was hosted in an online, low-risk, decentralised, non-clinical and direct to participant RCT. It is generalisable to other low-risk studies which use online recruitment and electronic participation information leaflets sent directly to participants via email. Additionally, this study also replicates the findings of studies using postal PILs. However, these findings may not apply to higher risk clinical studies where safety information needs to be conveyed and consent documented by a medical practitioner.

## Implications

This SWAT identified that recruitment and retention outcomes were not statistically significantly different between participants recruited to the IBD-BOOST RCT using a standard-length or shortened online PIL and replicates the findings of previous studies using postal PILs. These results suggest that a shortened PIL in a decentralised trial may have the same effect on trial recruitment and retention outcomes as a standard-length PIL, and more concise information could be sent to participants in future studies. This is in line with the UK HRA’s guidance that PILs should be proportionate and not go into too much detail, but rather ensure that a clear and concise picture of the research is given, providing that a trial intervention does not require extensive information in the PIL for other reasons (e.g. safety). Additionally, the shortened PIL did not generate more queries from potential participants, there was no cost difference as both PILs were online, and it was approved by the REC, once a shortened version of the HRA’s GDPR transparency wording was added. This suggests that RECs realise that length of standard PILs is a potential problem, though it should be noted that the shortened PIL did also include a link to the full information sheet which may have made it more acceptable.

Therefore, it can be argued that researchers have a choice about how long to make their PILs, and could consult with PPI members for their thoughts and feedback about what the best approach might be for a particular study. Furthermore, given that there was also no benefit of using a shortened PIL, it may be worth comparing written PILs with other methods of conveying study information to help determine the optimal means of encouraging participation and retention in decentralised trials. Alternatives such as staging approaches and/or multimedia formats could have the added benefit of encouraging participation from diverse groups who experience language or education barriers, or those with learning disabilities or cognitive impairments, which the NIHR’s INCLUDE guidance [20] was commissioned to address. The NIHR will also be making inclusive research design a condition of funding from autumn 2024 onwards [21]. As discussed earlier, difficulties with recruitment and retention can impact reliability, generalisability, statistical certainty, resource waste and access to potentially effective treatments, and it could be worth considering, as trials become more innovative, whether also a different approach to imparting participant information might be more effective.

## Supplementary Information

Supplementary Material 1.

## Data Availability

The host IBD-BOOST RCT is registered on the ISRCTN registry (ISRCTN71618461) and this SWAT is number 229 on the SWAT repository. The protocol for the IBD-BOOST RCT can be found using the following URL: https://osf.io/g9x3e, and the protocol for the SWAT is also available here: https://osf.io/amebx The data from the SWAT will be stored with the host PROMETHEUS programme at the University of York and can be requested from them.

## Abbreviations

CIs: Confidence intervals
CCUK: Crohn’s & Colitis UK
DCT: Decentralised clinical trial
GDPR: General Data Protection Regulation
IBD: Inflammatory bowel disease
MRC: Medical Research Council
NHS: National Health Service
OR: Odd ratios
PILs: Participant Information Leaflets
PPI group: Patient and public involvement
PDF: Portable Document Format
RCT: Randomised controlled trial
REC: Research Ethics Committee
SD: Standard deviation
SWAT: Study within a trial
TSC: Trial Steering Committee
HRA: UK Health Research Authority
NIHR: UK National Institute of Health and Care Research
NRES: UK National Research Ethics Service
